# Extracellular Vesicle/Macrophage Axis: Potential Targets for Inflammatory Disease Intervention

**DOI:** 10.3389/fimmu.2022.705472

**Published:** 2022-06-13

**Authors:** Desheng Tang, Feng Cao, Changsheng Yan, Kun Fang, Jiamin Ma, Lei Gao, Bei Sun, Gang Wang

**Affiliations:** ^1^ Department of Pancreatic and Biliary Surgery, The First Affiliated Hospital of Harbin Medical University, Harbin, China; ^2^ Central Laboratory, The First Affiliated Hospital of Harbin Medical University, Harbin, China; ^3^ Department of General Surgery, Xuanwu Hospital, Capital Medical University, Beijing, China; ^4^ Clinical Center for Acute Pancreatitis, Capital Medical University, Beijing, China

**Keywords:** macrophages, inflammation, extracellular vesicle, M1-like macrophage polarization, M2-like macrophage polarization

## Abstract

Extracellular vesicles (EVs) can regulate the polarization of macrophages in a variety of inflammatory diseases by mediating intercellular signal transduction and affecting the occurrence and development of diseases. After macrophages are regulated by EVs, they mainly show two phenotypes: the proinflammatory M1 type and the anti-inflammatory M2 type. A large number of studies have shown that in diseases such as mastitis, inflammatory bowel disease, Acute lung injury, and idiopathic pulmonary fibrosis, EVs promote the progression of the disease by inducing the M1-like polarization of macrophages. In diseases such as liver injury, asthma, and myocardial infarction, EVs can induce M2-like polarization of macrophages, inhibit the inflammatory response, and reduce the severity of the disease, thus indicating new pathways for treating inflammatory diseases. The EV/macrophage axis has become a potential target for inflammatory disease pathogenesis and comprehensive treatment. This article reviews the structure and function of the EV/macrophage axis and summarizes its biological functions in inflammatory diseases to provide insights for the diagnosis and treatment of inflammatory diseases.

## 1 Introduction

**Figure 1:**
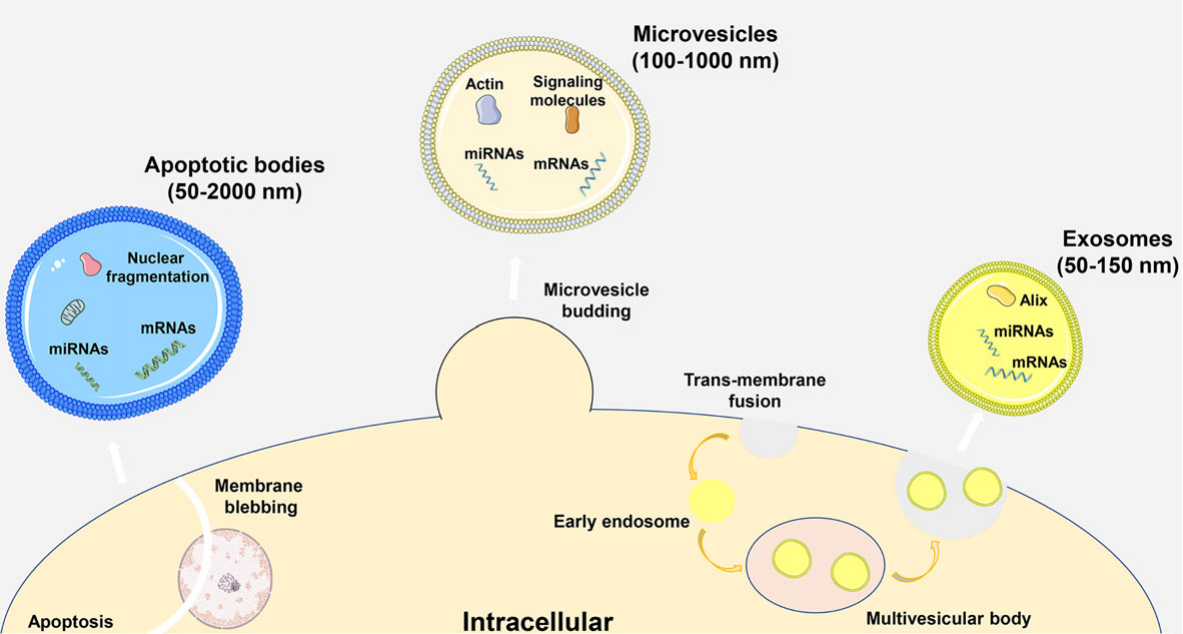
Particle size, formation process and classification of EVs.

Extracellular vesicles (EVs) are phospholipid bilayer structures secreted by cells. They contain lipids, proteins, nucleic acids and other components and participate in cell-to-cell communication by transmitting internal active substances and important mediators, thereby regulating the biological functions of recipient cells (1, 2). Traditionally, EVs were subgrouped into three main entities based on the pathway of production and particle diameter, including apoptotic bodies, microvesicles and exosomes. Apoptotic bodies are vesicles with a diameter of 50-2000 nm generated by the cell membrane wrapping the cytoplasm, DNA and organelles during apoptosis. Microvesicles are vesicles with a diameter of approximately 100-1000 nm produced by plasma membrane sprouting. Exosomes are vesicles with a diameter of 50-150 nm that are derived from multivesicular bodies formed by invagination of intracellular lysosomal particles and are released into the extracellular matrix after fusion of the outer membrane of multivesicles with the cell membrane (**Figure 1**) (3). In a variety of physiological or pathological conditions, EVs can be synthesized and secreted by different types of cells, and they are widely distributed in almost all body fluids. Especially in inflammatory diseases, secretory cells release more EVs to regulate the body’s immunity and metabolism. Recipient cells can take up EVs in a variety of ways, including direct membrane fusion, endocytosis, phagocytosis and micropinocytosis, and these methods are susceptible to changes in the body’s microenvironment (4). In particular, the first two methods play an important role in the process of information transmission, and phagocytosis is mainly involved in the metabolism of substances. As an important part of immune regulation, macrophages can be regulated by a variety of signals, including EVs, and are important target cells for regulating inflammation (5). Among the substances that transmit information in EVs, microRNAs (miRNAs) are the most widely studied. As noncoding single-stranded RNA molecules, miRNAs can bind to target genes and regulate the progression of inflammatory diseases by inhibiting gene expression. In particular, miRNAs can affect the immune microenvironment of the body by affecting the phenotype of macrophages. Therefore, exploring the composition and changes of miRNAs in EVs in disease is crucial for understanding the immunoregulatory functions of EVs. EV-miR-221 secreted by mammary epithelial cells can induce M1-like polarization of local mammary macrophages, thereby worsening mammary gland inflammation, while EVs derived from adipose-derived stem cells (ADSCs) can mediate M2-like polarization of pulmonary macrophages, thereby reducing lung inflammation and airway remodeling (6, 7). With the deepening of EV research, the characteristics of EVs, such as their high stability, high compatibility, and low immunogenicity, are gradually being recognized and applied to clinical research.

As an important part of the immune response process, macrophages play a pivotal role in the initiation, exacerbation and recovery of inflammatory diseases. The diversity of their functions depends on the high degree of plasticity of macrophages, which is a stable characteristic (8–12). Depending on the microenvironment and exogenous stimulus, polarization represents two activation states with different biological functions. Macrophage polarization is a complex process of multifactor interactions that is regulated by a variety of intracellular signaling molecules and pathways. In this process, when interferon-α (IFN-α), lipopolysaccharide (LPS) or tumor necrosis factor-α (TNF-α) activate Janus kinase/signal transduction and activator of transcription (JAK-STAT), Toll-like receptor/nuclear factor-kappaB (TLR-NF-κB) and other pathways, polarization is manifested as the M1 polarization (classical activation), which plays a proinflammatory function. However, when interleukin (IL)4, 13 (IL-4, IL-13) and other interventions activate phosphoinositide 3-kinase/proteinase-kinase B (PI3K-AKT) and other pathways, macrophages undergo M2 polarization (bypass activation), which exerts anti-inflammatory and tissue repair effects (8, 13, 14). M1-like macrophages in the intestine induce chronic inflammation of the intestine, which may induce the occurrence of inflammatory bowel disease, whereas M2-like macrophages in the skin help reduce skin inflammation and promote skin wound healing (15, 16). In addition, the polarization of macrophages is also affected by transcription factors, such as STAT1 and NF-κB is involved in the regulation of M1-like polarization of macrophage, while STAT3, STAT6 and Kruppel-like factor 4 (KLF4) are involved in the regulation of M2-like polarization of macrophage (17–20).

Although we mentioned the M1/M2 phenotype throughout the manuscript, this dichotomous description does not accurately reflect the multifunctional macrophages in the body. At present, some new types of macrophages such as CD169+ macrophages and T-cell receptor-positive macrophages, have also been found to regulate the immune system. Especially with the continuous progress of single-cell sequencing, more macrophages expressing special functions will be discovered to play an important role in different inflammatory diseases. Regarding the progression of the disease, we refer to the pathway through which EVs regulate the phenotype of macrophages as the EV/macrophage axis. Recent studies have shown that EVs play an important regulatory role in a variety of inflammatory diseases by regulating the different polarization types of macrophages, thereby prompting us to develop a more comprehensive understanding of the occurrence and development of inflammatory diseases. Thus, this article reviews the functions of EVs in the regulation of macrophage polarization in different inflammatory diseases.

## 2 EV/Macrophage Axis and Inflammatory Diseases

### 2.1 Mastitis

Acute mastitis is often caused by the blockage of the breast ducts caused by bacterial infections and other causes of acute inflammation of the breast tissue. If not treated in time, it may lead to the formation of breast abscesses (19, 21). In severe cases, sepsis may occur and even threaten the life of the patient. In the early stages of acute mastitis, breast epithelial cells secrete cytokines and chemokines, which attract inflammatory cells, such as surrounding macrophages. The NLRP3 inflammasome and NF-κB signaling pathways in macrophages are activated under the stimulation of various proinflammatory cytokines, which manifests as M1-like polarization and play a vital role in regulating the immune response of mastitis (22). MiR-221 has a proinflammatory effect in both vascular and pneumonia diseases (23, 24). Cai M et al. showed that EV-miR-221 derived from breast epithelial cells acts on suppressor of cytokine signaling 1 (SOCS1) to inhibit the expression of SOCS1. SOCS1 is an inhibitor of the JAK-STAT pathway, thus EV-miR-221 can lead to activation of JAK1. Downstream STAT1 is then phosphorylated and dimerizes into the nucleus, which induces the expression of M1-related marker genes, thereby promoting acute mastitis (6). The EV-miR-221/SOCS1/STAT1/STAT3 axis not only increases the proportion of M1-like macrophages in the early stage of mastitis but also provides a new direction for exploring the pathogenesis of mastitis.

### 2.2 Inflammatory Bowel Disease

Inflammatory bowel disease is a chronic inflammation of the intestines that mainly affects the ileum, rectum and colon, with abdominal pain and diarrhea as the main manifestations, which are caused by unknown mechanisms (25, 26). Although the pathogenesis is unclear, it is currently believed to be closely related to the destruction of immune cells. As one of the important effector cells in intestinal immunity, the intestinal macrophages of patients with inflammatory bowel disease have more M1-like phenotypes, producing a large number of proinflammatory cytokines to respond to the bacteria and playing an important role in maintaining the intestinal microenvironment (27, 28). Lee et al. showed that a high-fat diet is closely related to the occurrence of inflammatory bowel disease. Compared with the values obtained for the normal group, the blood levels of IL-6 and monocyte chemoattractant protein-1 (MCP-1) were significantly increased in the obese group, the inflammation of the intestinal tissue was greater, and the probability of inflammatory bowel disease was significantly increased (29). As one of the common proinflammatory miRNAs, miR-155 plays a role in almost all inflammatory diseases and affects the immune system by altering the production of inflammatory factors, such as TNF-α and interferon-β(IFN-β) (30). Studies by Wei et al. have proven that adipose tissue can not only secrete adipokines to affect the immune environment of the body but also secrete a large number of EVs into the blood and participate in the immune regulation of various organs throughout the body. A high-fat diet switches the EVs from an anti-inflammatory to a proinflammatory phenotype. Adipocytes secrete proinflammatory EV-miR-155 into the blood circulation, and after being taken up by macrophages in the intestine, macrophages undergo M1-like polarization, thus leading to chronic inflammation of the intestine (16). The EV-miR-155/macrophage axis provides a new basis for exploring the etiology of inflammatory bowel disease. In addition, EVs can be used for the treatment of inflammatory bowel disease. An et al. showed that EVs from ADSCs can effectively alleviate the severity of colitis. Tumor necrosis factor-α-stimulated gene/protein-6 (TSG-6) in EVs plays a key role in polarizing macrophages from M1-like to M2-like in the colon (31). Regulating the intestinal immune environment by reversing the polarization of macrophages will become an important method for the treatment of inflammatory bowel disease.

### 2.3 Inflammatory Diseases of the Lung

#### 2.3.1 Acute Lung Injury

Acute lung injury is an inflammation of acute lung parenchyma with dyspnea as the main phenotype. Acute respiratory distress syndrome (ARDS) can occur in severe cases and threatens the life and health of patients (32, 33). The disease is caused by many factors, and sepsis is an important factor. The lung is the most vulnerable organ for sepsis, and toxic substances in the circulating blood are likely to be the cause of Acute lung injury (34). In the early stages of acute lung injury, the activation of TLRs or other pattern recognition receptors (PRRs) triggered by infection immediately converts resident alveolar macrophages to an M1-like phenotype (35). Jiang et al. found that EV-miR-155 in circulating blood can activate the NF-κB level of lung macrophages by inhibiting the activity of SOCS1, which significantly increases the proportion of M1-like macrophages in the lungs of mice and aggravate Acute lung injury. In addition, EV

-miR-155 can also inhibit the expression of Src homology 2-containing inositol-5′-phosphatase 1(SHIP1) to promote the proliferation of macrophages and aggravate inflammation (36). In addition to EVs in circulating blood, EVs derived from neutrophils or lung epithelial cells are also involved in the regulation of acute lung injury. EV-miR-30d-5p derived from neutrophils induces M1-like polarization of macrophages in sepsis-related acute lung injury (37). Moon et al. showed that hyperoxia-induced lung epithelial cell-derived caspase-3-rich EVs activate proinflammatory macrophages and mediate inflammatory lung injury (38). On the other hand, EVs can also be used to relieve acute lung injury. Pulmonary macrophages are essential for the regulation of Acute lung injury, especially in terms of inducing the conversion of M1-like macrophages to the M2-like phenotype, which significantly improves the degree of inflammation in the lungs of patients (39).Tian et al. showed that EV-miR-16-5p from ADSCs promote the transformation of M1-like macrophages to an M2-like phenotype and attenuate septic lung injury by suppressing TLR4 (40). In addition, EVs from bone marrow mesenchymal stem cells (BMSCs) ameliorate lung inflammation and pathological damage by inhibiting glycolysis in macrophages to inhibit M1-like polarization and promote M2-like polarization (41). Generally, the function of EVs is closely related to the type of secretory cells and the immune environment. Regulating immunity through the influence of EVs on the phenotype of macrophages may become an important method for the treatment of Acute lung injury.

#### 2.3.2 Asthma

Asthma is a chronic specific airway inflammation characterized by reversible dyspnea, airway remodeling and bronchial hyperresponsiveness. The total number of macrophages in the lungs of asthmatic patients does not change significantly, but the proportion of M1-like macrophages increases significantly (42). M1-like macrophage can affect the production of inflammatory cytokines/chemokines, thereby regulating airway inflammation, and indicate that nitric oxide (NO) released by M1-like macrophages can cause deoxyribonucleic acid (DNA) damage to tracheal epithelial cells, resulting in an increase in the degree of airway inflammation and mucus secretion, which play a pivotal role in the onset of asthma (42, 43). Circular RNA (circRNA), as an important information transmitting substance in EVs, mostly binds to miRNAs to regulate the expression of NF-κB and other inflammation-related transcription factors, and it is involved in various inflammatory diseases, such as nephritis, pneumonia and vascular smooth muscle inflammation (44–46). Sang et al. showed that EV-mmu_circ_0001359 derived from ADSCs can enhance the forkhead box transcription factor O1(FoxO1) signaling pathway by inhibiting the expression of miR-183-5p and inhibit the M1 macrophage marker inducible nitric oxide synthase (iNOS), TNF-α and IFN-β in the airway and promotes the expression of M2 macrophage markers arginase 1 (Arg1) and Ym1, mediates the activation of M2-like macrophages, and significantly reduces the expression of proinflammatory factors and apoptosis of lung tissue cells alleviate lung fibrosis and airway remodeling, promote angiogenesis, and significantly improve lung inflammation (47). In addition, Dong et al. found that EVs derived from human umbilical cord mesenchymal stem cells can alleviate asthma by inducing the conversion of M1-like macrophages to an M2-like phenotype (48). Therefore, EVs can be an attractive candidate for the treatment of asthma by regulating the phenotype of macrophages.

#### 2.3.3 Idiopathic Pulmonary Fibrosis

Idiopathic pulmonary fibrosis is an unexplained chronic and progressive inflammatory disease of pulmonary interstitial fibrosis (49, 50). Due to the impaired systemic immune system and the unknown cause, specific medicinal treatments are not available for this disease. The continuous progress of stem cell research has shown that stem cells have anti-inflammatory effects and significantly improve lung fibrosis and lung function and can be used as a potential therapy for idiopathic pulmonary fibrosis (51). Compared with the normal control group, the number of M1-like macrophages in the lung lavage fluid of idiopathic pulmonary fibrosis patients was significantly increased, and the continuous inflammatory response served as a trigger to initiate the pulmonary fibrosis response, while inducing the polarization of M2-like macrophages improved the degree of pulmonary fibrosis (52). Tan et al. showed that the percentage of CD206+ (M2) macrophages was significantly higher and the percentage of CD86+ (M1) macrophages was lower following stimulation of macrophages with EVs from human aortic endothelial cells (hAECs). Therefore, EVs derived from hAECs induce the conversion of M1-like macrophages to an M2-like phenotype in pulmonary macrophages and increase macrophage phagocytosis. A bioinformatics analysis of EVs showed that miR-23a, miR-203a, miR-150 and miR-194 and other miRNAs were differentially expressed and thus may regulate the polarization of M2-like macrophages through PI3K-Akt and other pathways. The application of EVs not only reduces neutrophil myeloid peroxidation but also directly inhibits T cell proliferation and reduces lung inflammation and fibrosis (53). Therefore, these EV/macrophage axes are expected to become key targets for the treatment of Idiopathic pulmonary fibrosis.

### 2.4 Liver Inflammatory Lesions

#### 2.4.1 Cholestatic Liver Disease

Cholestatic liver disease is due to the accumulation of bile and the obstruction of the bile ducts, which results in abnormal metabolism of bile acids, inflammation of the entire hepatobiliary system, fibrosis and even liver failure in severe cases, including primary biliary cirrhosis and primary sclerosing cholangitis (54, 55). Guicciardi et al. found that compared with normal liver, peribiliary proinflammatory (M1-like) and alternate activation (M2-like) monocyte-derived macrophages in cholestatic liver disease increased, and peribiliary monocyte-derived macrophages underwent cell recruitment (M1-like>M2-like polarization) (56). Bile duct cells, as one of the cell types that secrete bile, are potential targets for the treatment of cholestatic liver disease. Long noncoding RNAs (lncRNAs) are abnormally expressed in rheumatoid arthritis, nephritis, bronchial asthma and other inflammatory diseases and often work by inhibiting the activity of miRNAs. LncRNAs in blood can also be used as molecular tools for highly sensitive and accurate disease diagnosis (57, 58). Li et al. showed that EV-LncH19 secreted by bile duct cells can promote the secretion of MCP-1 and IL-6 by macrophages, thereby promoting the activation and migration of more macrophages to the liver. In addition, EV-LncH19 also enhanced the expression of M1 macrophage markers [IL-6, IL-1β and cyclooxygenase-2 (Cox-2)], while the expression of M2 macrophage markers (IL-10) was not affected. Therefore, EVs are involved in chemokine-mediated inflammatory cell infiltration and liver inflammation and play a vital role in disease progression by inducing macrophages to differentiate into an M1-like phenotype (59). The EV/macrophage axis is expected to become a new pathogenesis of cholestatic liver disease. Interventions in each link of this axis may help us control the progression of cholestatic liver disease.

#### 2.4.2 Nonalcoholic Steatohepatitis

Nonalcoholic steatohepatitis is caused by the abnormal accumulation of lipids in liver cells, which causes liver cell damage and inflammation (60, 61). Excessive intake of fatty acids and lipid accumulation in the liver in nonalcoholic steatohepatitis promotes the activation of proinflammatory Kupffer cells and triggers the influx of peripheral blood macrophages into the liver, inducing chronic liver inflammation. The degree of inflammation in the liver is a key factor in the progression of nonalcoholic steatohepatitis, and increasing the activation of M1-like macrophages can promote the deterioration of nonalcoholic steatohepatitis. Tumor necrosis factor-related apoptosis-inducing ligand (TRAIL) is a member of the “tumor necrosis factor superfamily” (TNFSF), which can directly induce the activation of immune cells, such as macrophages, and thus plays a role in disease (62). Hirsova et al. showed that lipotoxic hepatocyte-derived EVs increased the expression of M1 markers in macrophages, especially TRAIL in EVs. This can promote the activation of macrophages to the proinflammatory M1-like phenotype by activating the NF-κB signaling pathway, leading to the secretion of a large number of proinflammatory cytokines and exacerbating liver damage (63). Other studies have shown that active integrin β1 (ITGβ1) in EVs derived from lipotoxic hepatocytes can also promote monocyte/macrophage adhesion and liver inflammation in nonalcoholic steatohepatitis (64). In addition, reducing the activation of M2-like macrophages can also worsen nonalcoholic steatohepatitis. Dasgupta et al. showed that nucleus signaling 1 (IRE1A) is activated in the liver of patients with nonalcoholic steatohepatitis and induces hepatocytes to release ceramide-rich inflammatory EVs. EVs can recruit macrophages to the liver and reduce alternatively activated M2-like macrophages, thereby aggravating the inflammation and damage of nonalcoholic steatohepatitis (65). The EV/macrophage axis provides a new direction for the pathogenesis of nonalcoholic steatohepatitis, and inhibiting or reducing the secretion of lipotoxic hepatocyte EVs is expected to be the next step in treatments involving the inhibition of nonalcoholic steatohepatitis inflammation progression.

### 2.5 Kidney Inflammation

Tubular interstitial inflammation is a common feature of acute and chronic kidney injury, and it affects renal function due to inflammatory infiltration and edema of the renal interstitium and is caused by different reasons (66, 67). In the initial phase of kidney injury, increased chemokines promote circulating monocyte chemotaxis into the kidney, developing into infiltrating Ly-6Chi macrophages exhibiting proinflammatory phenotype that participates in the regulation of renal inflammation (68). Renal tubular epithelial cells (TECs) are the key cells that regulate nephritis. After being stimulated by injury, they can secrete a large number of chemokines and cytokines to recruit inflammatory cells to the injured kidney, thus leading to aggravation of kidney inflammation (69). MiR-19b-3p is also related to a variety of inflammatory diseases. Circulating blood miR-19b-3p is not only positively correlated with the severity of knee osteoarthritis but can also regulate liver fat metabolism and inflammation pathways in patients with nonalcoholic steatohepatitis. In addition, it is also involved in promoting hypoxia-inducible factor 1alpha (HIF-1α) to regulate blood vessel inflammation (70). Li et al. showed that after TEC-derived EV-miR-19b-3p is internalized by macrophages, it targets SOCS1/NF-κB pathway, which causes M1-like polarization and the secretion of a large number of inflammatory factors, thus leading to the progression of kidney disease (71). In-depth studies of the EV-miR-19b-3p/SOCS1/NF-κB axis could provide new molecular targets for the further treatment of renal interstitial inflammation. In addition to miRNAs in EVs, chemokines and interleukins are also involved in the progression of the disease. Macrophage internalization of MCP-1 in EVs from bovine serum albumin (BSA)-treated TECs led to an enhanced inflammatory response and macrophage migration, which constitutes a critical mechanism of albumin-induced tubulointerstitial inflammation (69). EVs can also be used as a natural drug delivery system. EVs carrying IL-10 can effectively drive M2-like polarization to improve renal tubular damage and inflammation in tubular interstitial inflammation (72).

### 2.6 Cardiac Inflammatory Disease

#### 2.6.1 Myocardial Ischemia Reperfusion Injury

Myocardial ischemia-reperfusion injury is caused by myocardial ischemia, occurs because of local microcirculation disorder after myocardial infarction. After reperfusion, the myocardium releases a large number of proinflammatory factors and oxidative stress products to further aggravate myocardial tissue damage. The cascade inflammation after myocardial infarction is very important for the patient’s prognosis (73, 74). In the early period after myocardial ischemia-reperfusion injury, the number of cardiac macrophages increased rapidly. These initial infiltrative groups showed a proinflammatory phenotype, which in the following days changed to a repair phenotype, coordinating damage repair (75). EVs can affect the progression of myocardial ischemia-reperfusion injury by affecting the polarization of macrophages. Ge et al. found that miR‐155‐5p in EVs derived from the heart promoted macrophage M1-like polarization through the JAK2/STAT1 pathway and contributed to both local and systemic inflammation (76). In addition, Macrophages, especially M2-like macrophages, are essential in the process of myocardial ischemia-reperfusion recovery *via* the secretion of various cytokines and anti-inflammatory factors. Mesenchymal stem cells (MSCs) are a special type of pluripotent stem cell that can inhibit the expression of inflammatory factors, regulate the microenvironment of inflammation, reduce the area of myocardial infarction damage, improve heart function, and enhance angiogenesis. These cells are useful for the treatment of ischemic cardiomyopathy. Zhao et al. showed that MSC-derived EV-miR-182 is involved in mediating the transformation of M1-like macrophages to an M2-like phenotype by targeting the TLR4/NF-κB/PI3K/Akt signaling cascade and reducing myocardial infarct size and inflammation (77). In addition to myocardial ischemia-reperfusion injury, miR-182 also inhibits inflammation by acting on TLR4 during cerebral ischemia-reperfusion injury and spinal cord injury (73, 78). In addition to EV-miR-182, Shen et al. showed that EV-miR-21-5p is derived from MSCs and plays a similar role in myocardial ischemia-reperfusion injury (79). The EV/macrophage axis participates in the protection of the myocardium and the regulation of immunity, which provides a basis for the transplantation of MSC-EVs after myocardial infarction in the future.

#### 2.6.2 Dilated Cardiomyopathy

Dilated cardiomyopathy may be caused by the inflammation and apoptosis of cardiomyocytes that lead to remodeling of the heart. The main manifestations are the expansion of the ventricles and the decreased contractility of the myocardium (80, 81). In the myocardial injury stage of dilated cardiomyopathy, large numbers of macrophages are recruited to the injured site, accounting for 75% of all infiltrating cells. Under the influence of the cardiac microenvironment, macrophages are mainly polarized to an M1-like phenotype and participate in the immune response of diseases (82). Studies by researchers have applied echocardiography and hematoxylin-eosin (HE) staining of the heart, and the results showed that myocardial diastolic function was significantly improved, the proportion of apoptotic myocardial cells was significantly reduced, and the expression level of inflammatory factors was reduced in the MSC-derived EVs treatment group compared with the normal group. MSC-derived EVs could regulate the balance between M1-like and M2-like macrophages. MSC-derived EVs promote macrophage polarization toward an anti-inflammatory phenotype through the JAK2/STAT6 pathway, thus providing an anti-inflammatory environment for cardiomyocytes (83). The EV/macrophage axis regulates the polarization of macrophages, improves the inflammatory environment, blocks the development of dilated cardiomyopathy and heart remodeling, and provides a direction for the treatment of dilated cardiomyopathy.

### 2.7 Brain Inflammation

Spinal cord injury and traumatic brain injury are often caused by direct or indirect violence to the brain and spinal cord. In the early stage of the disease, sudden violence causes damage to the body, and subsequent cascade expansion of inflammation causes nerve cell necrosis. Axon destruction and demyelination increase the area of the lesion after injury and aggravate the degree of nervous system damage (84, 85). Therefore, controlling the degree of inflammation after injury has become a key link in the treatment of brain and spinal cord trauma. In the early stage of injury, both M1-like and M2-like microglia/macrophages are activated; in particular, a large number of M1-like macrophages activate the prolonged inflammatory phase and cannot quickly initiate repair. Therefore, adjusting the polarization balance of M1-like and M2-like macrophages has been shown to be essential for disease recovery (86, 87). Ni et al. showed that after traumatic brain injury, the application of BMSC-derived EVs not only inhibits the production of proinflammatory cytokines but also induces M2-like polarization of macrophages, reduces the degree of early inflammation, and reduces the area of the lesion after injury. BMSC-derived EVs exert a protective effect on the nervous system and could be used as a potential treatment strategy for the treatment of inflammation progression after traumatic brain injury in the future (88). Sun et al. believe that after spinal cord injury, the use of human umbilical cord mesenchymal stem cell-derived EVs can effectively induce bone marrow-derived macrophages (BMDMs) to transform the M1-like macrophage to M2-like phenotype and downregulate inflammatory cytokines to improve the microenvironment of inflammation after Spinal cord injury, thereby promoting spinal cord healing (89). Thus, BMSC-derived EVs could be used to develop future treatments for traumatic brain injury. In addition to the abovementioned BMSC-derived EVs, EVs, as natural drug carriers with high safety, can carry curcumin and berberine through the blood–brain barrier and improve the local microenvironment after brain injury by affecting the polarization of macrophages.

### 2.8 Vascular Inflammatory Diseases

#### 2.8.1 Abdominal Aortic Aneurysm

Abdominal aortic aneurysm is an inflammatory disease caused by the progressive expansion of blood vessels caused by multiple factors. Early inflammatory cells infiltrate the blood vessel wall and produce a large number of proinflammatory cytokines, which destroys the structure of smooth muscle and elastic fibers and weakens the wall of blood vessels. Sudden rupture of the blood vessel may then occur, which is the main cause of death (90, 91). Due to the lack of understanding of the pathogenesis of abdominal aortic aneurysm, effective drugs have not been developed for this disease; thus, treatment solely consists of surgery. Single-cell RNA sequencing reveals the simultaneous presence of proinflammatory and anti-inflammatory macrophages, which also means that inflammation and vascular repair both exist during the progression of abdominal aortic aneurysm. Therefore, the conversion of M1-like macrophages to an M2-like phenotype has become a potential treatment for abdominal aortic aneurysms (92). Studies have shown that MSCs can be used to prevent abdominal aortic aneurysm by reducing the production of proinflammatory cytokines (especially IL-17). Moreover, MSC-derived smooth muscle cells can induce the regeneration of an elastic matrix in abdominal aortic aneurysm, and promotes the recovery of blood vessels (93, 94). Related studies have shown that miR-147 can affect the progression of periodontitis by inducing changes in the phenotype of macrophages. Spinosa et al. showed that MSC-derived EV-miR-147 reduced the infiltration of proinflammatory cells (CD3+ T cells, M1-like macrophages and neutrophils), increased α-smooth muscle actin (α-SMA) expression and decreased elastic fiber destruction in blood vessels, thereby reducing aortic inflammation and vascular remodeling (95). Therefore, intervention of the miR-147/macrophage axis can reduce the possibility of inflammation and rupture, providing a molecular framework for the future clinical application of MSC-derived EVs (96).

#### 2.8.2 Atherosclerosis

Atherosclerosis is a chronic inflammatory disease of the vasculature caused by a variety of factors. Due to prolonged inflammation, the tube wall can harden and the lumen may narrow, thus causing ischemia and necrosis in the local blood supply area. Due to abnormal lipid metabolism, after macrophages engulf lipids, abnormal accumulation can become an important factor that generates plaques (97, 98). The significant impact of macrophages on atherosclerosis depends not only on the function of the different macrophage phenotypes but also on the relative ratio of different phenotypes in the plaque. When the plaque is dominated by M1-like macrophages, the area of the plaque is increased; in contrast, when it is dominated by M2-like macrophages, the plaque area is reduced and the stability of the plaque is increased (99). He et al. showed that oxidized low-density lipoprotein (ox-LDL) can affect the expression of miR-155 in vascular endothelial cells *in vivo*. Vascular endothelial cells secrete EV-miR-155 into macrophages, and enhance macrophage activation by shifting the macrophage balance from anti-inflammatory M2-like macrophages toward proinflammatory M1-like macrophages, thereby reducing the formation of atherosclerosis. The EV-miR-155/macrophage axis provides a new mechanism for the occurrence of atherosclerosis (100). For the treatment of atherosclerosis, the application of MSCs prevented lumen stenosis caused by plaque enlargement compared with the control group. Related studies have shown that M2-like macrophages can improve the apoptosis and lipid deposition induced by ox-LDL and promote the stability of atherosclerotic plaques (101). Li et al. showed that EVs derived from MSC treatment markedly inhibited LPS-induced M1 marker expression and increased M2 marker expression in macrophages. In particular, EV-miR-let7 regulates M2-like macrophage polarization and macrophage infiltration by targeting high-mobility-group protein AT-hook 2 (HMGA2) and insulin-like growth factor 2 mRNA-binding protein 1 (IGF2BP1). This inhibits the NF-κB pathway and reduces proinflammatory cytokines (TNF-α, IL-6 and IL-1β) to inhibit inflammation, thereby helping to reduce the area of atherosclerotic plaques (102). Regulating the EV-miR-let7/HMGA2/NF-κB axis can alleviate the inflammatory state of atherosclerosis and provides a new target for the treatment of atherosclerosis.

### 2.9 Obesity-Related Inflammation

Obesity is recognized as a risk factor for diseases affecting multiple systems of the body, including cardiovascular and endocrine diseases. Persistent low-level inflammation in adipose tissue is the basis of disease (103, 104). Adipose tissue contains a large number of immune cells that can secrete cytokines and inflammatory mediators into the blood circulation. Macrophages are important immune regulators and can play a role in monitoring and regulating inflammation (105). In the case of obesity, adipose tissue macrophages are polarized and transformed into a proinflammatory phenotype, which plays a key role in the early stages of obesity and in the maintenance of adipose tissue inflammation (106). Regulation of EVs is also an important part of maintaining M1-like macrophages in adipose tissue. Adipose tissue-derived retinol-binding protein 4 (RBP4) in EVs promotes inflammatory activation of macrophages through the TLR4/NF-κB pathway to regulate local or systemic immune metabolism homeostasis (107). Pan et al. found that adipocytes secrete EVs and significantly shift the polarization of adipose-resident macrophages from an anti-inflammatory M2-like phenotype to a proinflammatory M1-like phenotype. Among them, EV-miR-34a can inhibit the expression of M2-like macrophages by inhibiting the expression of Klf4 and can aggravate tissue inflammation (108). Therefore, the EV/macrophage axis is a new pathway for the production of obesity-related inflammation. In addition, when adipose tissue macrophages change from M1-like macrophages to an M2-like phenotype, a large number of anti-inflammatory cytokines are produced, which reduce the inflammation of adipose tissue. Relevant studies have shown that ADSC-derived EVs are transferred into macrophages to induce anti-inflammatory M2-like phenotypes through the transactivation of Arg1 by STAT3 and to help regulate diet-induced obesity and metabolic-related inflammation (7). ADSC-derived EVs also provide a new direction for the next step in the treatment of fat-related inflammation and reducing systemic complications.

### 2.10 Skin Inflammatory Diseases

Skin wound healing disorder is a serious complication of diabetes caused by the accumulation of macrophages and the release of proinflammatory cytokines, which leads to abnormal inflammation of the skin (109). Macrophages exposed to the diabetic environment mainly possess M1-like phenotypes, which are important for maintaining the skin’s proinflammatory environment. Hyperglycemia can reduce ability of macrophages to phagocytose, which can have a negative impact on the body’s clearance of apoptotic cells and will affect the body’s healing function. Therefore, coordinating the inflammatory response may be an important strategy to promote proper wound healing. MSCs can not only induce differentiation and promote skin regeneration but also inhibit inflammation, thereby promoting the recovery of skin damage. Ti et al. showed that MSC-derived EVs can promote the polarization of M2-like macrophages by upregulating the expression of anti-inflammatory factors, thereby regulating the balance of macrophages. In particular, EV-Let-7b can mediate the TLR4/NF-κB/STAT3/AKT signaling pathway to regulate the M2-like polarization of macrophages, reduce inflammation in diabetic patients and promote the prognosis of wounds (15). Kim et al. showed that human cord blood plasma EVs also promoted wound healing. Especially in EVs, heat shock protein 72 (HSP72) and prolactin-inducible proteins (PIPs) also promote differentiation from the proinflammatory M1-like phenotype to the anti-inflammatory M2-like phenotype, thus promoting the transition of wounds from inflammation to proliferation (110). Adjusting the EV/macrophage axis to reduce skin inflammation may become a new treatment approach for diabetes complicated with skin healing disorders in the future.

### 2.11 Acute Pancreatitis

Acute pancreatitis, one of the common clinical acute abdomens, is usually accompanied by local and systemic clinical symptoms. In particular, severe acute pancreatitis has a mortality rate of more than 20%, which seriously threatens the life of patients. Early in the disease, damage-associated molecular patterns (DAMPs) released by acinar cells can exacerbate pancreatitis by recruiting immune cells to the site of injury and activating an inflammatory response. Macrophages, especially M1 macrophages, are rapidly activated in the initial stage, leading to disease progression by releasing a large number of inflammatory factors and interleukins. Wu et al. showed that M1 macrophages predominate during the inflammatory progression phase of acute pancreatitis, while M2 macrophages predominate during pancreatic repair/regeneration (111). In addition to DAMPs, related studies have shown that EVs can also act as messaging tools to exacerbate acute pancreatitis. Laia et al. suggested that EVs in the circulating blood of patients with acute pancreatitis are one of the important substances that induce lung injury during acute pancreatitis (112). Wu et al. also confirmed that EVs in the circulating blood of patients with acute pancreatitis aggravated lung injury by inducing NLRP3-dependent pyroptosis of macrophages in the lung (113). In addition, EVs also play a crucial role in the treatment of pancreatitis. Wang et al. believe that Klotho-overexpressing MSC-derived EVs can reverse apoptosis and NF-κB activation in cerulein-stimulated AR42J cells, promising a potential treatment for acute pancreatitis (114). Therefore, the study of EVs is crucial for in-depth exploration of the pathogenesis and treatment of pancreatitis.

### 2.12 Sepsis

Sepsis is a dysfunctional systemic inflammatory disease caused by microbial infection, often accompanied by multiple organ failure. As one of the important cells of the innate immune response, macrophages play an important role in the immune regulation of patients with sepsis. It is well known that due to the state of immunosuppression, patients with sepsis often show the loss of immune defences and wound repair impairment. M2 macrophages are essential for the repair of immunosuppression, so the induction of M2 macrophage polarization helps to regulate the patient’s immune microenvironment (115). Ma et al. showed that endothelial progenitor cell-derived EVs can carry lncRNA taurine upregulated gene 1 and can promote M2 macrophage polarization and reduce the severity of sepsis by impairing miR-9-5p-targeted sirtuin 1(SIRT1) inhibition (116). In addition, MSC-derived EVs also have similar functions. Yao et al. believed that MSC-derived EV miR-21 could induce M2 macrophage polarization by inhibiting programmed cell death 4(PDCD4), thereby alleviating sepsis (117). In addition, reducing M1 macrophage polarization also alleviates sepsis. Bai et al. showed that adipose tissue-derived EVs can reduce M1 macrophage polarization and alleviate sepsis by modulating the Notch-miR148a-3p signalling axis (118). Therefore, a comprehensive understanding of the function of EVs will help to understand the pathogenesis of sepsis more comprehensively and establish a foundation for the treatment of sepsis in the future.

### 2.13 Diabetes Mellitus

**Figure 2:**
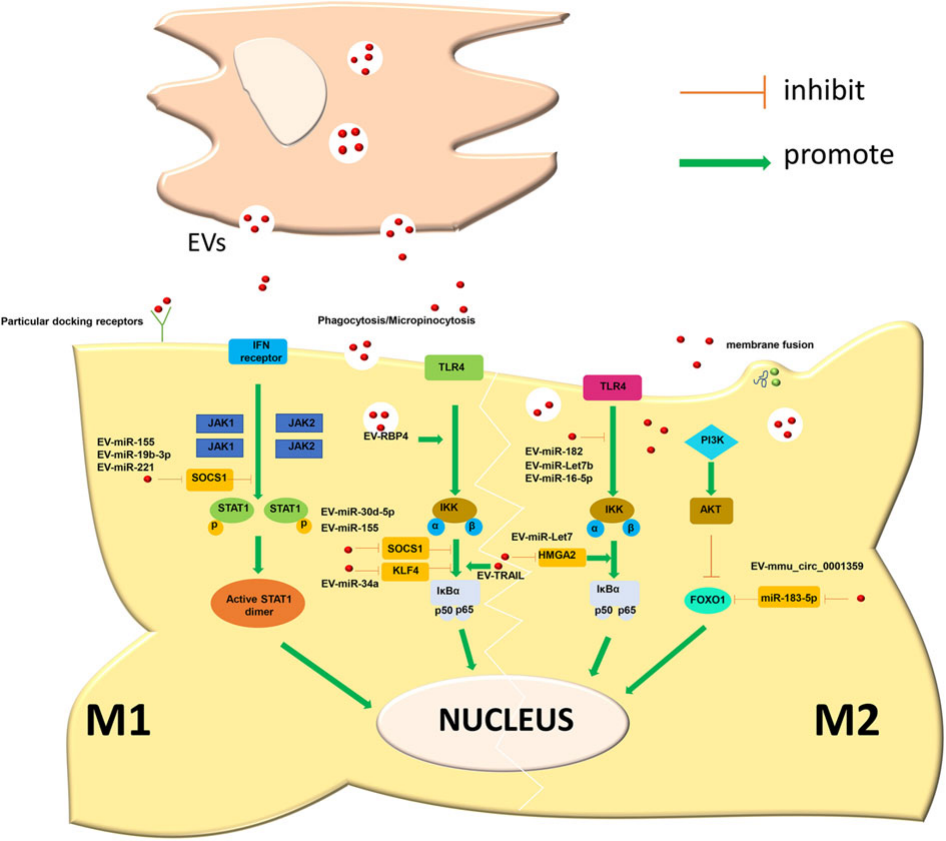
Secretory cells release EVs to induce macrophage polarization through a variety of ways.

Diabetes mellitus is a metabolic disease characterized by hyperglycaemia caused by multiple aetiologies. Long-term hyperglycaemia can lead to complications in multiple organs and tissues, such as the cardiovascular system, kidney and skin, affecting the quality of life of patients. Diabetes is mainly divided into four major types: type 1 diabetes, type 2 diabetes, special type diabetes and gestational diabetes. The most common clinical type is type 2 diabetes, and insulin resistance is the main cause of type 2 diabetes. Persistently high glucose in the internal environment induces an increase in M1-type macrophages and keeps systemic tissues in a state of persistent low-grade inflammation. Relevant studies have shown that EVs derived from the faeces of diabetic patients can be transplanted into mice and induce insulin resistance. Therefore, EVs may be an important factor leading to insulin resistance (119). Song et al. believed that adipocyte-derived EVs carry Sonic Hedgehog to induce M1 polarization of macrophages through the Ptch/PI3K pathway, leading to insulin resistance in adipocytes and inducing the occurrence and aggravation of diabetes (120). In addition to insulin resistance, EVs are also involved in the progression of diabetic nephropathy. Diabetic nephropathy is one of the devastating microvascular complications of diabetes and the most common cause of end-stage renal disease, with a mortality rate of 30-40%. Jia et al. showed that miR-199a-5p from human serum albumin (HSA)-stimulated HK-2 cell-derived EVs induced M1 polarization by targeting the Klotho/TLR4 pathway and further accelerated the progression of diabetic nephropathy (121). Therefore, an in-depth understanding of the role of EVs in diabetes can help us explore the pathogenesis of the disease. In addition, EVs have also shown promising effects in the treatment of diabetes-related complications. Delayed healing or nonunion of wounds caused by diabetes is a common problem encountered by clinical surgeons, and it has been difficult to solve thus far. Liu et al. showed that melatonin-pretreated MSC-derived EVs could upregulate the expression of PTEN and inhibit the phosphorylation of AKT to increase the ratio of M2/M1 cells and promote vascularization, *in vivo* production, and collagen synthesis, accelerating wound healing in diabetic patients (122). With the development of biotechnology, EVs are used for the treatment of diabetes in the clinic due to their unique advantages (**Figure 2** and **Table 1**).

**Table 1:** The role of EVs in macrophage polarization and pathological significance in inflammatory diseases.

	Source of EVs	EVs content	Particle size/markers	Macrophage cell type/phenotype	Major outcome	References
Mastitis	Mammary epithelial cells	miR-211	50 to 150 nm/CD81 and TSG-101	RAW264.7 macrophages/M1	MECs derived EV-miR-221 mediates M1-like macrophage polarization *via* SOCS1/STATs	Cai et al. (6)
Inflammatory bowel disease	Adipose tissue	miR-155	CD63 and TSG101	Macrophage from colon tissue/M1	Visceral adipose tissue derived EVs exacerbate colitis severity *via* promoting macrophage M1-like polarization	Wei et al. (16)
	Adipose-derived stem cells (ADSCs)	Tumor necrosis factor-α-stimulated gene/protein-6 (TSG-6)	less than 100 nm in diameter/CD63 and CD9	DH82 macrophage/M2	TSG-6 in EVs plays a key role in polarizing macrophage from M1-like to an M2-like phenotype	An et al. (31)
Acute lung injury	Peripheral circulating serum	miR-155	40-150 nm/CD63 and CD9	RAW264.7 macrophages/M1	Serum derived EV-miR-155 promote macrophage proliferation and inflammation by targeting SHIP1 and SOCS1 mediated M1-like macrophage polarization	Jiang et al. (36)
	Polymorphonuclear neutrophils	miR-30d-5p	70 nm/CD9, CD63 and TSG101	Raw264.7 macrophages or bone marrow-derived macrophages (BMDMs)/M1	EV-miR-30d-5p activates NF-κB in macrophage *via* targeting SOCS-1 and SIRT1	Jiao et al. (37)
	Lung epithelial cell	Caspase-3	50-120 nm/CD40L, integrin β1, Vps27, Vps32, Vps24, Vps4 and flot-1	Alveolar macrophages/M1	Lung epithelial cell-derived and caspase-3-rich EVs activate macrophages and mediate the inflammatory lung responses	Moon et al. (38)
	ADSCs	miR-16-5p	100 nm/CD9, CD63, and CD81	RAW246.7 macrophages/M2	EV-miR-16-5p from ADSCs promotes M2-like macrophage polarization *via* suppressing TLR4.	Tian et al. (40)
	Bone marrow mesenchymal stem cells (BMSCs)	–	80-150 nm/CD63 and CD81	MH-S macrophage/M2	BMSCs inhibited M1-like polarization and promoted M2-like polarization in MH-S cells by releasing EVs	Deng et al. (41)
Asthma Asthma	ADSCs	mmu_circ_0001359	CD63 and CD81	RAW264.7 macrophages/M2	ADSCs derived EV-mmu_circ_0001359 attenuate airway remodeling by targeting FoxO1 mediated M2-like macrophage polarization	Shang et al. (47)
	Human umbilical cord mesenchymal stem cell	–	107-217 nm/TSG101 and HSP70	RAW 264.7 macrophages/M2	MSC-EVs treatment inhibited M1-like polarization and promoted M2-like polarization in LPS-stimulated RAW 264.7 cells.	Dong et al. (48)
Idiopathic pulmonary fibrosis	Human amnion epithelial cell (hAEC)	miR-23a, miR-203a, miR-150 and miR-194	80-120 nm/Alix, CD81 and CD9	Macrophage from Lung lavage fluid/M2	hAEC derived EVs triggered the macrophage Polarization from M1-like to an M2-like phenotype	Tan et al. (53)
Cholestatic liver disease	Cholangiocyte	lncRNA H19	–	Primary Kupffer cells and BMDMs/M1	Cholangiocyte derived EV-lncRNA H19 promotes M1-like macrophage Activation	Li et al. (59)
Nonalcoholic steatohepatitis	Hepatocyte	Tumor necrosis factor-related apoptosis-inducing ligand (TRAIL)	40–300 nm with a mode size of 85 nm/Alix, TSG101 and ARF6	BMDMs/M1	Lipotoxic hepatocyte derived EVs activate M1-like macrophages in a DR5-dependent manner	Hirsova et al. (63)
	Hepatocyte	Integrin β1 (ITGβ1)	110-300 nm/TSG101, CD63 and CD81	BMDMs/M1	Lipotoxic hepatocyte-derived EVs are enriched with active ITGβ1, which promotes monocyte adhesion and liver inflammation in murine NASH.	Gao et al. (64)
	Hepatocyte	Ceramide	150-200 nm/TSG101, Alix, CD81 and CD9	BMDMs/decrease M2	IRE1A-treated hepatocyte-derived EVs enriched in ceramides reduce M2-like macrophage polarization.	Dasgupta et al. (65)
Kidney inflammation	Tubular epithelial cells (TECs)	miR-19b-3p	61.2-128.8 nm/Alix, CD9 and CD63	Raw 264.7 and BMDMs/M1	TECs derived EV-miR-19b-3p promotes M1-like macrophage polarization through targeting SOCS-1/NF-κB	Lv et al. (71)
	TECs	Monocyte chemoattractant protein-1 (MCP-1)	59.5-147.5 nm/Alix, CD9 and CD63	RAW264.7/M1	Macrophage internalization of MCP-1 in EVs from BSA-treated TECs led to an enhanced inflammatory response and macrophage migration	Lv et al. (69)
	RAW264.7	Interleukin-10 (IL-10)	10-500 nm, with a mean diameter of 134 nm/Alix, CD63 and CD81	RAW264.7/M2	IL-10^+^ EVs efficiently drive M2-like macrophage polarization, which can suppress inflammation and promote kidney repair.	Tang et al. (72)
Myocardial ischemia reperfusion injury	Heart	miR-155-5p	10-400 nm/CD63, CD9, Alix and TSG101	BMDMs and peritoneal macrophages/M1	Heart derived miR‐155‐5p in EVs promotes M1-like macrophage polarization through activating JAK2/STAT1 pathway	Ge et al. (76)
	MSCs	miR-182	50-150 nm, with mode of 142 nm/CD63, CD9, TSG101, and Alix	RAW264.7 macrophages/M2	MSCs derived EV-miR-182 mediated macrophage polarization by targeting the TLR4/NF-κB/PI3K/Akt pathway.	Zhao et al. (77)
	MSCs	miR-21-5p	CD63, CD9, TSG101 and Alix	RAW264.7 macrophages/M2	MSCs derived EV-miR-21-5p promotes the polarization of RAW264.7 cells to an M2-like phenotype	Shen et al. (79)
Dilated cardiomyopathy	MSCs	–	35.21 nm/N-Alix, TSG101, CD9 and CD63	Macrophages in both blood and heart/M2	MSCs derived EVs relied on the JAK2-STAT6 pathway mediating macrophages activation	Sun et al. (83)
Traumatic brain injury (TBI)	MSCs	–	110.4 nm/TSG101 and CD63	Macrophages from brain/M2	MSCs derived EVs decreased the activation of macrophage M1-like phenotype but increased M2-like phenotype after TBI	Ni et al. (88)
Spinal cord injury	MSCs	–	70 nm/CD63, CD81, CD9 and TSG101	BMDMs/M2	MSCs derived EVs triggered the macrophage polarization from M1-like to an M2-like phenotype	Sun et al. (89)
Abdominal aortic aneurysm	MSCs	miR-147	–	Macrophages were purified from spleens/M2	MSCs derived EV-miR-147 down-regulated IL-17 and HMGB-1 expression promoting M2-like macrophage activation	Spinosa et al. (95)
Atherosclerosis	Aortic endothelial cells	miR-155	100 nm/CD63	THP-1 cell line/M1	Endothelial derived EV-miR-155 mediates M1-like macrophage polarization	He et al. (100)
	MSCs	miR-let7	70-100 nm	Macrophages were induced from U-937 cells/M2	MSCs derived EV-miR-let7 promotes M2-like macrophage polarization and macrophage infiltration by targeting HMGA2 and IGF2BP1	Li et al. (102)
Obesity-related inflammation	Adipose tissue	Retinol binding protein 4(RBP4)	60-100 nm	BMDMs/M1	Adipose tissue derived RBP4 in EVs promote inflammatory activation of macrophages through TLR4/NF-κB pathway.	Deng et al. (107)
	Adipocyte	miR-34a	30–100 nm/CD63	BMDMs/M1	Adipocyte derived EV-miR-34a mediates M1-like macrophage polarization by repressing the expression of Klf4	Pan et al. (108)
	ADSCs	Signal transduction and activator of transcription 3 (STAT3)	100 nm/TSG101, CD9, CD63 and HSP90	Peritoneal macrophages/M2	ADSC derived EVs drive M2-like phenotype polarization through transporting STAT3	Zhao et al. (7)
Skin inflammatory diseases	MSCs	miR-let-7b	40-90 nm/CD9, CD63 and CD81	THP-1 cell line/M2	MSCs derived EV-miR-let-7b regulates M2-like macrophage polarization *via* TLR4/NF-κB/STAT3/AKT signaling	Ti et al. (15)
	Human cord blood plasma	Heat shock protein 72 (HSP72) and prolactin-inducible proteins (PIPs)	69.72-185.46 nm/CD81, ICAM, CD63, ALIX, TSG101, EpCAM and FLOT-1	Human bone marrow cells/M2	Human cord blood plasma EVs promoted differentiation from the proinflammatory M1-like phenotype to the anti-inflammatory M2-like phenotype	Kim et al. (110)
Spesis	Endothelial progenitor cells (EPCs)	lncRNA Taurine upregulated gene 1(TUG1)	30-120 nm/Alix, TSG101, and CD9	RAW264.7 macrophages/M2	EPCs derived EVs transmitted *TUG1* to promote M2-like macrophage polarization through the impairment of miR-9-5p-dependent SIRT1 inhibition	Ma et al. (116)
	MSCs	miR-21	60.8-151.2 nm/CD63 and Alix	BMDMs/M2	MSCs-derived EV -miR-21 could induce M2-like macrophage polarization by inhibiting PDCD4	Yao et al. (117)
	adipose tissue	miR148a-3p	48.53-98.77 nm/CD9, CD63 and CD81	RAW264.7 macrophages/decrease M2	adipose tissue-derived EVs can reduce M1 macrophage polarization and alleviate sepsis by modulating the Notch-miR148a-3p signalling axis	Bai et al. (118)
	Adipocyte	Sonic Hedgehog (Shh)	67.07 nm/CD63, CD81 and TSG101	BMDMs/RAW246.7 macrophages/M1	Adipocyte-derived EVs carrying Shh induce the M1-like polarization of macrophages through the Ptch/PI3K pathway.	Song et al. (120)
Diabetes mellitus	HK-2 cells	miR-199a-5p	48.5-176.9 nm/CD63 and TSG101	THP-1 cells/M1	miR-199a-5p from HSA-stimulated HK-2 cell-derived EVs induces M1-like polarization by targeting the Klotho/TLR4 pathway	Jia et al. (121)
	MSCs	–	120 nm/CD81, TSG101 and Alix	RAW264.7 macrophages/M2	MSC-derived EVs induce the M2-like polarization of macrophages by targeting the PTEN/AKT pathway	Liu et al. (122)

## 3 Mechanism by Which EVs Induce Polarization of Macrophages

### 3.1 JAK-STAT Pathway

The JAK-STAT pathway is one of the most common pathways that induces macrophage polarization. STAT is a huge family of transcription factors, and when JAK is activated, STAT is phosphorylated into dimers (123, 124). Due to the reduced affinity of STAT, it dissociates and enters the nucleus to regulate the expression of related genes. Different STAT subtypes have different regulatory functions. For example, STAT6 is closely related to the polarization of M2-like macrophages. After JAK2 is stimulated by cytokines, it activates STAT6 to undergo dimerization into the nucleus, induces the expression of M2-related genes, promotes the activation of M2-like macrophages, and inhibits inflammation (20). In dilated cardiomyopathy, MSC-EVs can activate STAT6, thereby promoting M2-like macrophages to improve myocardial inflammation and heart remodeling. However, STAT1 is different from STAT6. When JAK1 is activated, it induces the activation of STAT1 in a similar manner, thereby promoting the activation of M1-like macrophages and promoting the aggravation of the inflammatory response (125). EV-miR-221 secreted by mammary epithelial cells in mastitis and cardiac-derived EV-miR-155-5p in myocardial ischemia-reperfusion injury can induce STAT1 activation and induce M1-like macrophage polarization to aggravate the disease.

### 3.2 TLR-NF-κB Pathway

The TLR family is the main receptor that recognizes various antigens on the surface of macrophages. When stimulated by EVs, it activates the IκB kinase (IKK) complex, which leads to phosphorylation of inhibitor of NF-κB (IκB) and proteasome degradation. The transcription factor NF-κB acts as a heterodimer, and it is released into the nucleus and induces the production of interleukins, cytokines and other substances, which induce macrophages to undergo a polarization reaction (126). In addition, this pathway also plays an important role in infectious diseases and autoimmune diseases (126, 127). Activation of this pathway promotes polarization of M1-like macrophages. EV-miR-30d-5p derived from polymorphonuclear neutrophils, EV-miR-155 in peripheral circulating serum in acute lung injury, EV-TRAIL derived from hepatocytes in nonalcoholic fatty liver disease and EV-RBP4 and EV-miR-34a derived from fatty tissue can induce the activation of NF-κB, which can promote M1-like polarization and aggravate the inflammatory response. However, inhibition of this pathway will convert M1-like macrophages to an M2-like phenotype. EV-miR-182 and EV-miR-let7 derived from MSCs can improve the progression of inflammation by inhibiting the NF-κB pathway in myocardial ischemia-reperfusion injury, atherosclerosis and skin inflammation. In addition, EV-miR-16-5p derived from ADSCs in acute lung injury can also improve lung inflammation by inhibiting this pathway.

### 3.3 P13K-AKT Pathway

**Figure 3:**
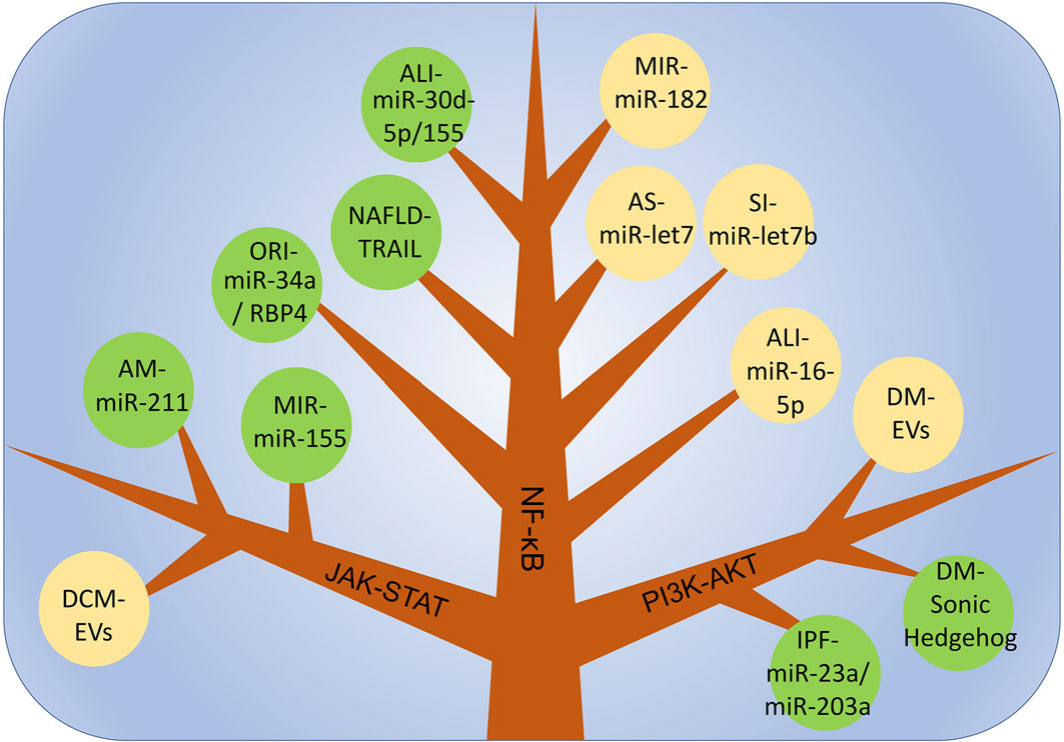
The main mechanism of macrophage polarization is in various inflammatory diseases. *DCM*, dilated cardiomyopathy; *AM*, acute Mastitis; *MIR*, myocardial ischemia-reperfusion injury; *ALI*, Acute lung injury; *NAFLD*, non-alcoholic fatty liver disease; *ORI*, Obesity-related inflammation; *SI*, Skin inflammation; *AS*, Atherosclerosis; *IPF*, Idiopathic pulmonary fibrosis; *DM*, Diabetes mellitus.

PI3K is an intracellular phosphatidylinositol kinase. When interacting with receptors or connexins and tyrosine residues, dimerization will occur and then downstream AKT will be phosphorylated. thereby induces the expression of downstream genes, which affects the polarization of macrophages. Different AKT subtypes are activated and affect the phenotype of macrophages. AKT1 activation promotes the differentiation of macrophages into the M2-like phenotype, and AKT2 induces macrophages to differentiate into the M1-like phenotype. Therefore, the PI3K-ATK pathway plays an important regulatory role in the polarization process in macrophages (128, 129). In idiopathic pulmonary fibrosis, miR-23a, miR-203a, miR-150 and miR-194 in EVs derived from hAECs promote the conversion of M1-like macrophages to an M2-like phenotype and improve inflammation by activating the PI3K-AKT pathway. In addition, this pathway is also closely related to the regulation of tumor cell proliferation, migration, adhesion, tumor angiogenesis and extracellular matrix degradation(**Figure 3**) (130).

## 4 Discussion and Prospects

With the in-depth study of EVs and macrophage phenotypes, the function of the EV/macrophage axis in the pathogenesis and treatment of inflammatory diseases has gradually been recognized. A large number of studies have shown that macrophages in a polarized state are critical to the progression of inflammatory diseases, and the current methods used to alter macrophage polarization and affect inflammation can be roughly divided into two categories (131). One method is to directly infuse M1 or M2 macrophages, especially M2 macrophages, which have been shown in the literature to control the progression of inflammation and promote tissue repair. However, the direct infusion of M2 macrophages may lead to immune rejection, and many experiments have produced controversial results; thus, its effectiveness cannot be confirmed (132). The other method is to interfere with inflammation by inducing the polarization of macrophages *in vivo*. The existing results confirm that its effect is better than that of the direct input of polarized macrophages. Based on the high plasticity of macrophages, appropriate interventions can be performed. The two phenotypes of macrophages can be reversibly converted in different cytokine environments to achieve the effect of regulating inflammation. In particular, the EV/macrophage axis can regulate the degree of inflammation in a variety of diseases and has broad prospects in the field of inflammation treatment.

With the help of high-throughput sequencing technology, we can comprehensively understand the changes in each component of EVs, which provides a direction for exploring the function of EVs. Among the current studies, miRNAs in EVs are the most widely studied. miR-155 is abnormally elevated in EVs in inflammatory bowel disease, acute lung injury, myocardial ischemia reperfusion injury and atherosclerosis and induces M1 macrophage polarization to promote inflammatory progression. miR-155 is an important proinflammatory mediator in a variety of diseases and aggravates the degree of inflammation in the disease. Therefore, the exploration of high-efficiency and specific miR-155 inhibitors may play an important role in the treatment of inflammation in the future. In addition, the most involved pathway in the EV/macrophage axis is the TLR-NF-κB pathway. As a classic M1-polarized pathway, activation of this pathway promotes the deterioration of inflammation, while blocking this pathway accelerates the body’s repair. Therefore, inhibitors of the pathway can block EV-mediated disease progression, providing a direction for future research on the clinical application of EVs. Clinical treatment targeting EVs has become a hot spot of current research. In almost every inflammatory disease, MSC-EVs inherit the anti-inflammatory and immunomodulatory properties of stem cells. They induce M2 polarization of macrophages, reduce inflammation, and avoid the disadvantages of stem cells, such as tumorigenicity. They will become a powerful weapon for the treatment of inflammatory diseases.

Compared with IL-4, IL-10 and miRNA, which conventionally induce M2 polarization, the EV/macrophage axis has unique advantages: (1) the specificity of the EV/macrophage axis. Because EVs can be specifically recognized by recipient cells, the antigen-antibody reaction on the surface is more accurate than miRNA or IL-10 and other substances that induce macrophages to polarize and can avoid binding to other nontargeted cells (133). (2) Low cytotoxicity and low immunogenicity of the EV/macrophage axis. For example, the high expression of substances such as IL-4 in the blood can cause asthma and other allergies or immune diseases and are not suitable as candidates for inducing macrophage polarization to treat inflammation (134); moreover, certain EVs have limited effects on the immune system and do not induce systemic reactions, especially MSC- EVs. MSC-EVs have been widely used, and the transplantation of MSC-EVs has shown good results in many animal models of inflammatory diseases, thus providing a theoretical basis for future clinical applications. (3) Manipulability of the EV/macrophage axis. With the development of modern bioengineering technology and the progress of nanomaterials, the surface or content of EVs has been modified to greatly increase the sensitivity of EV detection and the diversity of the contents. After the modified EVs enter the human body with extremely high biocompatibility, they can be accurately targeted to the location of damage. The process of EV release can be controlled by changes in certain signals, such as temperature or key proteins such as Rab27a (135). Such processes increase the manipulability of EVs and increases the flexibility of adjusting the phenotype of macrophages. However, many aspects of the EV/macrophage axis are still unknown at present, and there are some problems that need to be solved, such as the following. (1) The complexity of the EV/macrophage axis regulation. Although research on the EVs has made considerable progress in recent years, due to the difficulty in purifying EVs and constant changes in content with changes in the body environment, such work has not reached the level of clinical application. (2) Uncertainty in the regulation of the EV/macrophage axis. The same EV-miRNA regulates the phenotype of macrophages in different diseases. For example, EV-miR-147 causes M2-like polarization in abdominal aortic aneurysm, but induces M1-like polarization in periodontitis disease models (96). EV-miR-21 also has a similar performance. The reason for this phenomenon is not clear but may be related to the different sources of EVs in the experiment, the different sources of macrophages, or the different stages of the disease. Therefore, the same EV/macrophage axis plays different roles in different inflammatory diseases, which increases the complexity of using the EV/macrophage axis for treatment. (3) The complexity of EVs. EVs contain many nucleic acid and protein components. Are these different substances synergistic or antagonistic? Which substances play more important roles? Will the same substance perform the same function in different stages of the disease? These issues are yet to be explored. Therefore, to employ the EV/macrophage axis to treat inflammation, we must comprehensively consider various factors, thoroughly analyze the components of EVs, accurately regulate the entire pathway, and conduct individualized treatments.

## 5 Summary

The EV/macrophage axis plays an important role in the occurrence and development of inflammatory diseases and not only provides a direction for exploring the pathogenesis of inflammatory diseases ut also a new way to treat inflammatory diseases. As the regulation of the EV/macrophage axis becomes increasingly precise, EV treatments may be used as a therapeutic strategy for inflammatory diseases in the future and thus have broad prospects.

## Author Contributions

DT and FC involved in concept and writing of the manuscript; GW and BS revised the manuscript; CY, JM, KF, and LG made the tables and created the figures. All authors contributed to the article and approved the submitted version.

## Funding

This paper was supported by grants from the National Nature Scientific Foundation of China (No: 82070657 and No:81770639), Applied Technology Research and Development Plan of Heilongjiang Province in China (NO: GA20C019), Outstanding youth funds of the first affiliated hospital of Harbin Medical University (HYD2020JQ0006) and Research projects of Chinese Research Hospital Association (Y2019FH-DTCC-SB1).

## Conflict of Interest

The authors declare that the research was conducted in the absence of any commercial or financial relationships that could be construed as a potential conflict of interest.

## Publisher’s Note

All claims expressed in this article are solely those of the authors and do not necessarily represent those of their affiliated organizations, or those of the publisher, the editors and the reviewers. Any product that may be evaluated in this article, or claim that may be made by its manufacturer, is not guaranteed or endorsed by the publisher.

## Abbreviations

IFN-α, interferon-α; IFN-β, interferon-beta; LPS, lipopolysaccharide; TNF-α, tumor necrosis factor-α; JAK-STAT, janus kinase/signal transduction and activator of transcription; TLR-NF-κB, toll-like receptor/nuclear factor-kappaB; PI3K-AKT, phosphoinositide 3-kinase/proteinase-kinase B; IL, interleukin; KLF4, kruppel-like factor 4; IκB, inhibitor of NF-Kb; IKK, IκB kinase; SOCS1, cytokine signaling 1; MCP-1, monocyte chemoattractant protein-1; miRNAs, microRNAs; TSG-6, tumor necrosis factor-α-stimulated gene/protein-6; ARDS, acute respiratory distress syndrome; PRRs, pattern recognition receptors; SHIP1, src homology 2-containing inositol-5′-phosphatase 1; ADSCs, adipose-derived stem cells; BMSCs, bone marrow mesenchymal stem cells; NO, nitric oxide; DNA, deoxyribonucleic acid; circRNA, Circular RNA; FoxO1, forkhead box transcription factor O1; iNOS, inducible nitric oxide synthase; Arg1, arginase 1; hAECs, human aortic endothelial cells; lncRNAs, long noncoding RNAs; Cox-2, cyclooxygenase-2; TRAIL, tumor necrosis factor-related apoptosis-inducing ligand; TNFSF, tumor necrosis factor superfamily; ITGβ1, integrin β1; IRE1A, nucleus signaling 1; TECs, tubular epithelial cells; HIF-1α, hypoxia-inducible factor -1alpha; BSA, bovine serum albumin; MSCs, mesenchymal stem cells; HE, hematoxylin-eosin; ox-LDL, oxidized low-density lipoprotein; α-SMA, α-smooth muscle actin; BMDMs, bone marrow-derived macrophages; HMGA2, high-mobility-group protein AT-hook 2; IGF2BP1, insulin-like growth factor 2 mRNA-binding protein 1; RBP4, retinol-binding protein 4; HSP72, heat shock protein 72; DAMPs, damage-associated molecular patterns; PIP, prolactin-inducible proteins; SIRT1, sirtuin 1; PDCD4, programmed cell death 4; HAS, human serum albumin.
